# The effect of interfraction prostate motion on IMRT plans: a dose‐volume histogram analysis using a Gaussian error function model

**DOI:** 10.1120/jacmp.v10i4.3055

**Published:** 2009-09-30

**Authors:** James C. L. Chow, Runqing Jiang, Daniel Markel

**Affiliations:** ^1^ Department of Radiation Oncology Princess Margaret Hospital University Health Network Toronto ON Canada; ^2^ University of Toronto and Radiation Medicine Program Princess Margaret Hospital University Health Network Toronto ON Canada; ^3^ Department of Physics Ryerson University Toronto ON Canada; ^4^ Department of Physics University of Waterloo Waterloo ON Canada; ^5^ Medical Physics Department Grand River Regional Cancer Center Kitchener ON Canada

**Keywords:** dose‐volume histogram, prostate IMRT, internal organ motion, error function and model fitting

## Abstract

The Gaussian error function model, containing pairs of error and complementary error functions, was used to carry out cumulative dose‐volume histogram (cDVH) analysis on prostate intensity‐modulated radiation therapy (IMRT) plans with interfraction prostate motion. Cumulative DVHs for clinical target volumes (CTVs) shifted in the anterior‐posterior directions based on a 7‐beam IMRT plan were calculated and modeled using the Pinnacle^3^ treatment planning system and a Gaussian error function, respectively. As the parameters in the error function model (namely, *a*, *b* and *c*) were related to the shape of the cDVH curve, evaluation of cDVHs corresponding to the prostate motion based on the model parameters becomes possible, as demonstrated in this study. It was found that deviations of the cDVH for the CTV were significant, when the CTV‐planning target volume (PTV) margin was underestimated in the anterior‐posterior directions. This was especially evident in the posterior direction for a patient with relatively small prostate volume (39 cm^3^). Analysis of the cDVH for the CTV shifting in the anterior‐posterior directions using the error function model showed that parameters $a_{1,2}$, which were related to the maximum relative volume of the cDVH, changed symmetrically when the prostate was shifted in the anterior and posterior directions. This change was more significant for the larger prostate. For parameters *b* related to the slope of the $\text{cDVH},\;b_{1,2}$ changed symmetrically from the isocenter, when the CTV was within the PTV. This was different from parameters *c* ($c_{1,2}$ are related to the maximum dose of the cDVH), which did not vary significantly with the prostate motion in the anterior‐posterior directions and prostate volume. Using the patient data, this analysis validated the error function model, and further verified the clinical application of this mathematical model on treatment plan evaluations.

PACS number: 87.10.‐e, 87.55.‐x, 87.55.dk and 87.56.N‐

## I. INTRODUCTION

Dose‐volume histograms (DVHs) have been widely used to represent the 3D dose distributions on a 2D graph during radiotherapy treatment planning.^(^ ^1^ ^,^ ^2^ ^)^ When DVHs were included in treatment planning systems (TPSs), they became a useful tool in treatment plan evaluation. A DVH provides dose level and uniformity information for regions of interest such as the target or critical organs. The biological measures of tumor control probability (TCP) and normal tissue complication probability (NTCP) are calculated using the differential DVH (dDVH).^(^ ^3^ ^,^ ^4^ ^)^ For plan optimization of intensity‐modulated radiation therapy (IMRT), DVH control points in the cost function are used in the process of inverse planning optimization to determine the appropriate dose distribution.^(^ ^5^ ^–^ ^7^ ^)^ Although DVHs are a useful tool in 3D treatment planning evaluation and optimization, they cannot provide spatial dose information. Therefore, dose contour maps are still necessary for plan evaluations. Another shortcoming of DVHs is that voxels used in the histogram calculation are assumed to contain the same amount of equifunctioning cells focusing on the dose in volume rather than by mass. This limits the accuracy in evaluating radiobiological treatment plans.^(^ ^8^ ^–^ ^10^ ^)^ Regardless of their shortcomings, DVHs are still widely used for plan evaluations, calculations of TCP and NTCP, and inverse plan optimization using a commercial TPS.

For treatment plan evaluations using DVHs, an assessment of DVH curves through a quantitative inspection is sometimes needed to compare two curves (e.g. DVH curves for planning target volume (PTV)) with very similar slopes or shapes from separate IMRT plans. In these circumstances, it is desirable to convert the shape of the curve into quantitative parameters. The intention being that using this model to describe DVH characteristics can make planning evaluations more efficient. A curve‐fitting algorithm using a mathematical model was necessary for determining the parameters associated with a DVH curve. Van den Heuvel^(11)^ proposed a model based on a Gaussian normal distribution function to decompose a dDVH for analysis. This model allows for a quantitative approach to using a DVH by defining the dose delivered to a target in a more rigorous fashion. Recently, Chow et al.^(12)^ proposed another mathematical model based on the Gaussian error and complementary error functions for cumulative DVH (cDVH) analysis. In mathematics, the Gaussian error function is a nonelementary function of sigmoid shape. The error function, encountered when integrating a normal distribution, is very similar to a typical cDVH curve from an external beam treatment plan. The model of Chow et al. uses the relationship that a cDVH and an error function are integrals of a dDVH and a Gaussian normal distribution function taken from particular limits. Application of the error and complementary error functions together to fit the cDVH curve has been proven to decrease the number of parameters when modeling.^(12)^ However, such a mathematical model was only described using a rigid prostate phantom, and further validation of the model using clinical patient data was necessary.

Although prostate IMRT is well known to provide highly conformal doses to the target, and spare surrounding critical organs such as the bladder and rectum,^(^ ^13^ ^–^ ^15^ ^)^ a patient's interfraction prostate motion would cause uncertainty in the daily dose delivered to the target and critical organs during the course of treatment.^(^ ^16^ ^–^ ^18^ ^)^ In fact, the effects of prostate motion have been studied extensively by many groups. Langen et al.^(^ ^19^ ^,^ ^20^ ^)^ investigated the dosimetric effect of prostate motion in helical tomotherapy, and found that the change in the target $D_{95\%}$ due to prostate motion was generally small. Kerkhof et al.^(21)^ studied variations of target and rectal dose due to prostate deformation using MR imaging. They found that increases in rectal dose depended on the variation in intrapatient rectum position during treatment, but was negligible with current fractionated IMRT. Cranmer‐Sargison^(22)^ focused on the potential dosimetric effects of systemic rotational setup errors on prostate patients, as per the RTOG P‐0126 protocol. For patient setup error and prostate motion, Zhu et al.^(23)^ found that the current prostate IMRT protocol could successfully compensate for systematic uncertainties. While for intrafraction prostate motion, Li et al.^(24)^ found that a CTV‐PTV margin of 2 mm or greater would produce an insignificant dosimetric effect due to intrafraction prostate motion. However, the focus of this study is the application of the Gaussian error function in modeling cDVH curves. Three prostate patients with relatively small, medium and large prostates were selected from a group of 20 patients, and treatments were planned using a 7‐beam IMRT technique. Cumulative DVHs of the target and critical organs were calculated for each patient using the Pinnacle^3^ TPS (Philips Medical Systems, Andover, MA) while shifting the CTV in the anterior‐posterior directions. The error function model was used to carry out cDVH analyzes for these IMRT plans. Model parameters for each cDVH were determined, and their relationship to CTV displacement was studied. Moreover, the correlations between the change in TCP for CTVs with prostate motion, NTCPs for the bladders and rectums, and the corresponding prostate volume were studied. The aims of this study are to demonstrate the evaluation of a prostate IMRT plan through cDVH analysis based on the error function model for patients with different prostate volumes, and to prove that the model can be used for both phantom and clinical data.

## II. MATERIALS AND METHODS

### A. Patient data

Three patients with small, medium and large prostates were selected from a group of 20 prostate cancer patients being treated under the 7‐beam IMRT protocol at the Grand River Regional Cancer Center at Grand River Hospital. Information relating to patients’ prostate, rectum and bladder volumes is shown in Table 1(a).

**Table 1(a):** Patients’ information of prostate, rectum and bladder volumes.

*Patient*	*Prostate Volume (cm^3^)*	*Rectum Volume (cm^3^)*	*Bladder Volume (cm^3^)*
Small Prostate (SP)	39.0	52.0	471.6
Medium Prostate (MP)	60.0	71.5	114.7
Large Prostate (LP)	87.0	130.0	117.5

### B. Treatment planning

**Figure 1:**
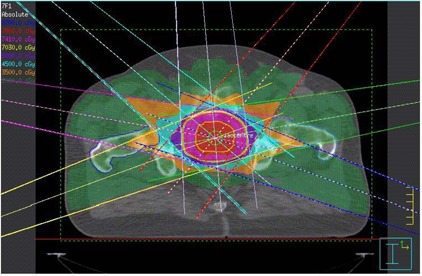
Diagram showing the beam geometry and dose distribution for the 7‐beam IMRT for the patient with a medium prostate volume.

For each patient's data (obtained with the same acquisitional parameters as when CT scanning), the prostate, seminal vesicle, rectum, bladder, left and right femur were contoured. The gross target volume (GTV) and CTV are replications of the prostate volume, and the PTV was created using the CTV with a 0.5 cm margin. For each patient, a 7‐beam IMRT plan was created using the Pinnacle^3^ TPS. Fifteen MV coplanar photon beams produced by a Varian 21 EX linear accelerator were used with beam angles of 40, 80, 110, 250, 280, 310 and 355 degrees, as shown in Fig. 1. A 120‐leaf Millennium multileaf collimator system was used to generate field segments for the beam intensity modulation. The prescription dose was 78 Gy with 2 Gy per fraction. The dose‐volume control points relating to target volumes and critical organs for the inverse planning can be found in Table 1(b). The minimum and maximum dose to the target volume as well as maximum dose to the critical organs are parameters in the optimization cost function. Also included in these parameters are the specified fractions of volume that are allowed to exceed the prescribed dose limit in the case of a critical organ, or in case of targets, to be less than the prescribed value. The relative weights of the beams were adjusted by changing DVH control points and penalties for the PTV and critical organs of the IMRT plan.

**Table 1(b):** DVH control points for the CTV, PTV, rectum, bladder, left and right femur for the 7‐beam IMRT plan.

Volume of Interest	DVH Control Point (cGy)
CTV	$D_{99}\;\geq \;7800$
PTV	$D_{99}\;\geq \;7410$
PTV	Maximum dose to ${1\;\text{cm}}^{3}\;\leq \;8190$
Rectum	$D_{30}\geq 7000$
Rectum	$D_{50}\geq 5430$
Bladder	$D_{30}\geq 7000$
Bladder	$D_{50}\geq 5430$
Left and Right Femur	$D_{5}\geq 5430$

When the IMRT plan based on the center of the PTV (i.e. the isocenter) was completed, cDVHs for CTV, bladder and rectum were calculated using the TPS. To simulate interfraction prostate motion, the CTV was shifted by 1 cm in the anterior and posterior directions in 2 mm steps using a dose distribution based on the IMRT plan without prostate motion. Therefore, in total 10 cDVHs were generated according to CTVs shifted from the isocenter (i.e. 10, 8, 6, 4 and 2 mm in the anterior direction, and 10, 8, 6, 4 and 2 mm in the posterior direction). In this study, the CTV displacement only focused on the anterior and posterior directions because it is well known that the prostate motion is most significant along the anterior‐posterior axis.^(^ ^25^ ^,^ ^26^ ^)^ Moreover, the CTV was moved out of the PTV, and the 0.5 cm margin (CTV‐PTV) used here underestimated the interfraction prostate motion. That is, the simulated motions were larger than what is anticipated clinically by the CTV to PTV expansion. This range of CTV displacement was selected because variations in the cDVH become more pronounced allowing a better demonstration of the error function model.^(12)^ It should be noted that the cDVH for the CTV did not vary significantly when it was moved within the PTV with sufficient margin in a treatment plan. All cDVH data for the IMRT plan with different CTV positions were exported from the Pinnacle^3^ TPS to MATLAB for model fitting.

### C. TCP and NTCP calculation

The TCP was calculated using a logistic regression equation

(1) $$\text{TCP}=\frac{\text{exp}(p+qD)}{1+\text{exp}(p+qD)}$$

where *D* is dose, *p* and *q* are related to $D_{50}$ and $\gamma_{50}$ (normalized slope at the point of 50% control probability). Okunieff et al.^(27)^ summarized clinical data for a variety of tumors and reported parameters that can be related to the slope and dose to control 50% of tumours. Equation 1 assumes that the TCP for the tumor irradiated to a given dose *D* is independent of the tumor size and, for this study, it is a valid assumption. Prostate volume does not affect the TCP directly. However, it does affect the IMRT dose distribution within the prostrate, specifically the dose gradient near the edges of the PTV. This in turn changes the behavior of the TCP when prostate motion occurs. Control probability for the tumorlet with volume and dose, $\text{TCP}(v_{i},\;D_{i})$ can be inferred from the TCP for the whole volume by the following:

(2) $$\text{TCP}(v_{i},D_{i})=\text{TCP}{\left(D_{i}\right)}^{v_{i}}$$

where $\left(D_{i},\;v_{i}\right)$ refers to the dDVH. TCPs calculated from the dDVHs for the CTVs were plotted against the CTV displacement in the anterior‐posterior directions.

NTCP is calculated using the Lyman‐Burman‐Kutcher algorithm^(^ ^28^ ^–^ ^30^ ^)^

(3) $$\text{NTC}P=\frac{1}{2\pi }\int_{-\infty }^{t}e^{-\frac{x^{2}}{2}}dx$$

and

(4) $$t=\frac{D-TD_{50}(v)}{mTD_{50}(v)}$$

where $v=V/V_{\text{ref}}$ and ${\text{TD}}_{50}(v)={\text{TD}}_{50}(1)\;v^{-n}$, (as suggested by Burman et al.^(28)^) a ${\text{TD}}_{50}$ of 80 Gy, an *n* of 0.12, and *m* of 0.15 were used for rectum here. Both TCP and NTCP in this study were determined using an in‐house TCP/NTCP calculator by first converting the cDVH to dDVH.^(30)^

### D. Error function model

The details of the theory of the error function model can be found in Chow et al.^(12)^ In short, the error and complimentary error function can be shown in the following equation:

(5) $$\text{DVH}(V)=a_{1}\;\text{erf}[b_{1}\cdot (D-c_{1})]+a_{2}\;\text{erf}c[b_{2}\cdot (D-c_{2})]$$

where $a_{1},\;b_{1,}$ and $c_{1}$ are parameters for the error function and $a_{1},b_{2}$ and $c_{2}$ are parameters for the complimentary error function. *D* and *V* are the dose and volume, respectively. In Eq. 5, the parameters, $a_{1,2},b_{1,2}$ and $c_{1,2}$ for the error and complimentary error function have their own characteristics in modeling the cDVH curve. The parameters $a_{1,2}$ vary with the maximum relative volume of the cDVH curve. The slope of the cDVH after the curve drop‐off can be adjusted with $b_{1,2}$, while varying $c_{1,2}$ will change where the cDVH drops off from the normalized volume close to 1. It should be noted that such relationships only hold when the parameter pairs are varied by the same amount. As the complimentary error function is just a subtraction of 1 from the error function, the characteristics of $a_{2},b_{2}$ and $c_{2}$ are similar to $a_{1},b_{1}$ and $c_{1}$. The pairing of a complimentary error function to an error function produces a “tailoring effect” for Eq. 5 to model the cDVH curve.

To obtain the fit parameters, Eq. 5 was fitted using the ‘cftool’ graphical user interface that is included in the curve fitting toolbox for MATLAB 7.1 (R14) service pack 3. Curves were fitted using combinations of the Trust‐Region and Levenberg‐Marquardt optimization algorithms for nonlinear least squares minimization. Initial coefficient values were estimated through small deviations of the optimized values for previous curves of similar shape. Manipulations of the initial values were mostly trial and error but guided by knowledge of how the variables determined the overall shape of the curve, as described in Chow et al.^(12)^ Optimal coefficients were judged based on R‐squared values, which fell above 0.94 for the majority of solutions. The optimal coefficient values were then themselves fitted, with the displacement of the organ being the independent variable. Using either a basic linear equation or a logarithmic one, pairs of variables (e.g. $a_{1}$ and $a_{2}$) were plotted against one another to determine relationships between the two and thus further reduce the degrees of freedom for possible solutions.

## III. RESULTS

**Figure 2:**
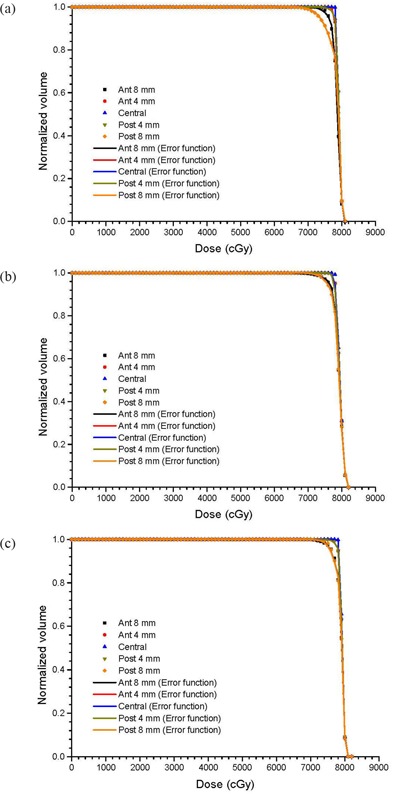
Cumulative DVHs for CTVs based on the 7‐beam IMRT plan using the TPS and error function model. The CTV is positioned at the isocenter and shifted 4 and 8 mm in the anterior and posterior directions for the patients with (a) small, (b) medium, and (c) large prostate volumes.

**Figure 3:**
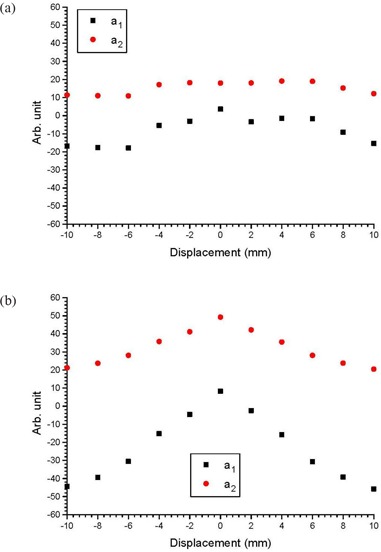
Parameters $a_{1}$ and $a_{2}$ varying with the shifted distance of the CTV in the anterior‐posterior directions for the patients with (a) small and (b) large prostate volumes.

**Figure 4:**
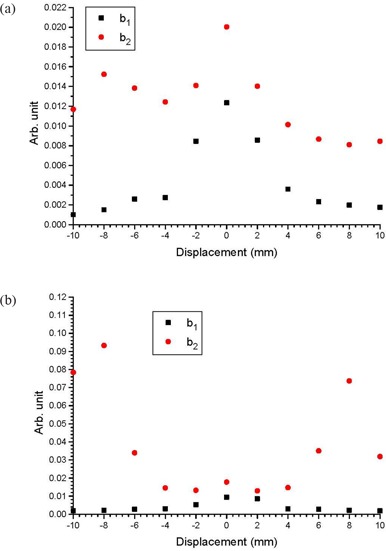
Parameters $b_{1}$ and $b_{2}$ varying with the shifted distance of the CTV in the anterior‐posterior directions for the patients with (a) small and (b) large prostate volumes.

**Figure 5:**
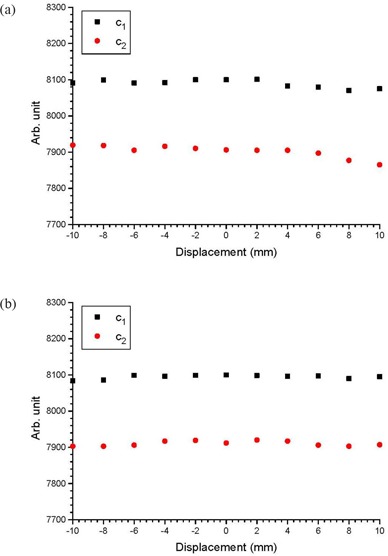
Parameters $c_{1}$ and $c_{2}$ varying with the shifted distance of the CTV in the anterior‐posterior directions for the patients with (a) small and (b) large prostate volumes.

**Figure 6:**
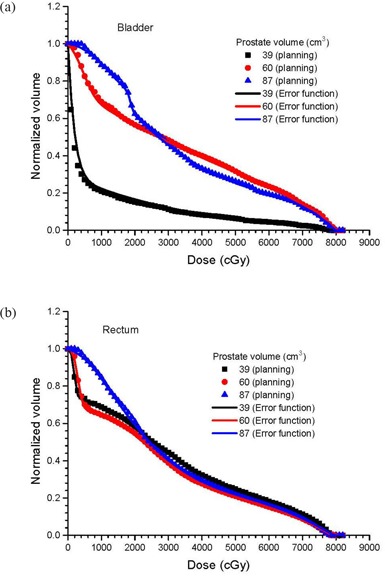
Cumulative DVHs for (a) bladders and (b) rectums based on the 7‐beam IMRT plan using the TPS and the error function model for the patients with small, medium, and large prostate volumes. The CTV is positioned at the isocenter.

**Figure 7:**
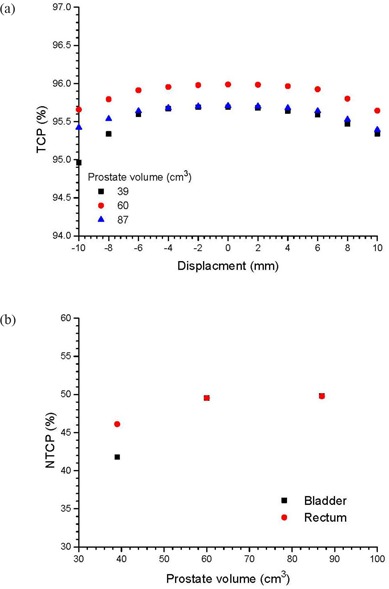
TCP varying with the shifted distance of the CTV for the patients with small, medium and large prostate volumes (a); NTCPs of bladders and rectums varying with the prostate volume (b).

Figures 2(a), 2(b) and 2(c) show cDVHs of the CTVs at the isocenter and other positions in the anterior‐posterior directions based on the same IMRT plans using the centers of the PTV as isocenters. This was done for three patients with small, medium and large prostates, respectively. To simplify the presentation, only cDVHs of the CTVs that shifted with distances of 4 mm and 8 mm anteriorly and posteriorly were plotted against cDVHs of the CTVs at their respective isocenters. Cumulative DVHs modeled by the error function are also plotted in Fig. 2. Figures 3(a) and 3(b) show the relationship between the parameters $a_{1,2}$ and CTV displacement for the patients with small and large prostates. Similarly, Figs. 4 and 5 plot the parameters $b_{1,2}$ and $c_{1,2}$ against the CTV displacement. Figures 4(a) and 5(a) are for the patient with a small prostate, while 4(b) and 5(b) are for the patient with a large prostate. The prostate volumes for the patients can be found in Table 1(a). The cumulative DVHs for the bladders and rectums of the three patients based on the IMRT plans with no prostate motion are shown in Figs. 6(a) and 6(b). The error function model was used to carry out curve fitting of the cDVHs in Fig. 6, and the results are shown in the same figure. Due to the more complex shape of the cDVHs for critical organs (Fig. 6) compared to the PTV/CTV (Fig. 2), a larger number of parameters were needed in the error function modeling. The results of fitting parameters in Fig. 6 for the three patients are shown in Table 1(c). Figure 7(a) shows TCPs for the CTVs shifted in the anterior‐posterior directions for the patients with small, medium and large prostates. The variations in NTCP for bladders and rectums versus the prostate volume are shown in Fig. 7(b).

**Table 1(c):** All fitting parameters of bladders and rectums for the patients with small, medium and large prostate volume using the error function model.

*Parameters*	*Bladder*			*Rectum*		
	*SP*	*MP*	*LP*	*SP*	*MP*	*LP*
$a_{1}$	2632	14.19	3.448	4.791	12.09	−15.12
$a_{2}$	216.3	156.6	124.4	52.63	64.51	105.5
$a_{3}$	2630	−2.24	−0.4401	0.1217	0.5938	−42.19
$a_{4}$	2628	−7.32	−14.44	−1.945	−4.824	−14.19
$a_{5}$	−	−	−22.62	−	17.13	−
$a_{6}$	−	−	−1.682	−	−16.74	−
$b_{1}$	−0.00157	−0.0024	−0.035	−0.01768	−0.00598	−0.00154
$b_{2}$	0.00015	7.94E‐05	0.000201	0.000118	5.14E‐05	0.000318
$b_{3}$	−0.00358	0.005956	2.218	−0.02495	1.678	0.000323
$b_{4}$	−0.00357	−0.00061	−0.00084	−0.00172	−0.00078	0.005977
$b_{5}$	−	−	−0.00105	−	0.000406	−
$b_{6}$	−	−	−8.536	−	−0.00057	−
$c_{1}$	−720.1	468.3	1820	177.7	302	78.14
$c_{2}$	−2339	−4039	−609.6	−740.4	−740.3	404.2
$c_{3}$	7677	7693	7803	7802	7802	8899
$c_{4}$	7677	7395	7800	7815	7793	20130
$c_{5}$	−	−	−275.8	−	−79.04	−
$c_{6}$	−	−	1987	−	2055	−

## IV. DISCUSSION

### A. Phantom and patient data

In Fig. 2, when the CTV is shifted from the isocenter in the anterior and posterior directions, the dose conformation of the prostate decreases. This decrease in dose conformation is proportional to the shifted distance of the CTV. This result agrees well with our prostate phantom case.^(12)^ Although a 5‐beam IMRT plan was used for the prostate phantom, the cDVHs of the CTVs that shifted at different positions (8 and 4 mm from the isocenter in the anterior‐posterior directions) are similar to those in Fig. 2. It shows that the rigid prostate phantom used to demonstrate the error function model is a valid approximation of patient data. Moreover, in Fig. 2 it can be seen that the cDVHs of the CTVs that shifted 4 mm to the anterior or posterior direction are very similar to the cDVHs of the CTVs without shifting. This is because a PTV defined by the CTV plus a 5 mm margin was used in the IMRT plan optimization. Therefore, if the CTV is not moved out (e.g. 4 mm) of the PTV, in theory the cDVH of the CTV should not change significantly compared to a case without prostate motion. However, if the CTV is moved 8 mm (> CTV−PTV margin=5 mm) out of the PTV, the cDVHs (e.g. “Ant 8 mm” and “Post 8 mm”) will be different from the ones with no CTV shift (as in Fig. 2). Comparing Figs. 2(a), 2(b) and 2(c), the cDVHs of the CTVs that shifted 8 mm in the anterior and posterior direction are very similar for Figs. 2(b) (medium prostate) and 2(c) (large prostate). However, for the patient with a small prostate (shown in Fig. 2(a)), the cDVHs of the CTVs that shifted 8 mm in the anterior and posterior direction are different. It is seen that the cDVH that shifted 8 mm in the posterior direction has poorer target dose conformation than that of the anterior. This is because the effect of the steep dose gradient at the prostate‐rectum interface for the small prostate (more rounded) is more significant than those for the medium and large prostate.^(30)^ The corresponding cDVHs modeled using the error function are also plotted in Fig. 2, and agree well with the cDVHs calculated by the TPS. The parameters, *a*, *b* and *c* (as mentioned in the Materials and Methods section) for the patients with a small and large prostate are shown in Figs. 3, 4 and 5.

### B. Variations of error function parameters with prostate motion and volume

Figures 3(a) and 3(b) show the relationship between parameters $a_{1,2}$ and CTV displacement. As parameter *a* is related to the maximum relative volume of the cDVH, it can be seen that such variation with the prostate motion is more significant for a larger prostate volume. This is due to the tailoring effect of the error and complimentary error functions. While the maximum volume of the large prostate does not vary significantly with displacement, the interplay of the amplitudes of the two error functions does. Moreover, both $a_{1}$ and $a_{2}$ were found to vary similarly with respect to the CTV displacement. Figures 4(a) and 4(b) show the variation of the parameters $a_{1,2}$ in relation to CTV displacement. Since parameter *b* is closely related to the slope of the cDVH after the curve drop‐off, and $b_{1}$ (error function component) is more dominant than $b_{2}$ (complementary error function component) in the cDVH modeling,^(12)^ it is seen that the cDVH of the CTV has the steepest slope (i.e. highest dose conformation at the target) when there is no prostate motion. This is because at zero CTV displacement, the values of $b_{1}$ in Figs. 4(a) and 4(b) are largest. With regard to the correlation between $b_{1}$ and $b_{2}$, it is interesting to note that $b_{2}$ does not match $b_{1}$ in Fig. 4(a), when the CTV is shifted out of the PTV in the posterior direction. This mismatch is more significant for a larger prostate volume (Fig. 4(b)), when both values of $b_{2}$ in the anterior and posterior direction outside the PTV do not follow the same trend as values of $b_{1}$. These mismatches cause a blunt drop‐off from the maximum relative volume, when the CTV is moved out of the PTV. This shows that when the CTV‐PTV margin is underestimated, prostate motion would decrease dose conformation around the CTV. Figures 5(a) and 5(b) plot the relationship between the parameter *c* and the prostate motion for the patients with a small and large prostate. It can be seen that both $c_{1}$ and $c_{2}$ do not vary significantly with prostate motion. As parameter *c* is related to the position at which the cDVH starts to drop off, Fig. 5 shows that the position of the drop‐off for the cDVH does not vary with prostate motion and volume.

### C. Error function model for cDVHs of critical organs

Figures 6(a) and 6(b) show the cDVHs of the bladders and rectums for the three patients. It can be seen in Fig. 6 that the dose‐volume relationships for the rectum in the medium and high dose regions (i.e. 30–80 Gy) are very similar and independent of the prostate volume. For the error function modeling, a larger number of parameter pairs (i.e. two or three instead of one compared to the CTV case) were required in the curve fitting, due to the more complex shape of the cDVH curve. The values calculated for the parameters *a*, *b* and *c* in Fig. 6 based on the error function model can be found in Table 1(c). Since a larger number of parameters were involved in the modeling of the cDVH of a critical organ than for the CTV, cDVH analysis for the critical organ using error function parameters becomes more difficult. However, good agreement between the cDVHs obtained from the TPS and the error function approximations show that Eq. 5 can be used to model cDVHs for critical organs with more complex shapes. An image deformation model using the cubic‐B‐spline deformable registration algorithm is under construction to study interfraction motions of the bladder and rectum.^(31)^ Related results will be presented in the future.

### D. Variations of TCP and NTCP with prostate motion and volume

The TCP and NTCP models provide a quantitative plan evaluation of a given dose distribution expressed in terms of dDVHs. Calculations of the TCP and NTCP are based on radiobiological models (such as that mentioned in Sec. II. C. above). When TCP and NTCP values are determined, the acceptance of the plan can be justified by comparing the TCP of the target and NTCPs of the critical organs. Generally, a large TCP value for the target (prostate), and small NTCP values for the critical organs are desired in a good IMRT plan.^(32)^

The variations in the TCP for the CTV with prostate motion can be found in Fig. 7(a) for the patients with small, medium and large prostates. In Fig. 7, it is seen for each patient that the maximum TCP is found when the CTV is not shifted. When the CTV is shifted to the anterior or posterior direction within the PTV, the TCP does not change significantly. For the small, medium and large prostates, the TCP only decreases by 0.05%, 0.02% and 0.03% when the CTV is shifted by 4 mm in the posterior direction. However, it is obvious that when the CTV is shifted outside the PTV (i.e. >5mm), the TCP decreases significantly if the prostate volume is small. The reason is partially due to a larger percentage of the volume lying outside the PTV of a given shift for small targets as compared to large ones. For example, considering a plane located 5 mm above a sphere with a radius of 2.1 cm representing the PTV, and a corresponding prostate volume of 39 cm^3^, a shift of 1 cm results in 4% of volume above the plane. However, when the prostate volume is increased to 87 cm^3^ (radius = 2.75 cm), the same distance of shift results in a smaller volume of 2.3% above the plane. In the medium and large prostate cases, shifting the CTV 10 mm in the posterior direction only decreases the TCP by 0.34% and 0.29%, respectively. However, the TCP decreases by 7.7% when the CTV is shifted by the same distance (10 mm) and direction (posterior) for the small prostate. This demonstrates that a smaller prostate is more sensitive to target dose coverage in our 7‐beam IMRT plan. In Fig. 7(b), the NTCPs for bladders and rectums increase with the prostate volume. Therefore, though the patient with a small prostate has a similar TCP as the one with a large prostate, the overall IMRT plan for the patient with a small prostate is better, because NTCPs for the bladder and rectum are lower.

## V. CONCLUSIONS

The error function model, pairing the Gaussian error and complementary error function, was used to analyze cDVHs of the CTV and critical organs (bladder and rectum) in several prostate IMRT plans. Seven‐beam IMRT plans were created for three patients with small, medium and large prostates using a CTV‐PTV margin of 5 mm. Interfraction prostate motion was simulated by shifting the CTV from 2 mm to 10 mm in the anterior and posterior directions in 2 mm steps, with new cDVHs calculated at each step for the CTV. Similar to the prostate phantom case in our previous work,^(12)^ this study involved verification of the error function model using clinical patient data. It is found that the fitting parameters, *a*, *b* and *c*, corresponding to various characteristics of the cDVH curves for the CTV, are useful in analyzing the dose‐volume relationship. This was demonstrated by investigating relationships between model parameters of the cDVH and interfraction prostate motion. It is concluded that the error function model of the cDVH for the CTV provides an alternative view of IMRT planning evaluation. The model has potential to further study certain issues in prostate radiotherapy, such as PTV‐CTV margin optimization within a certain confidence level, and the marker‐based localization technique.
